# Beyond Accuracy: A Mixed-Methods Audit of Chain-of-Thought Failures in LLM-Based COVID-19 Vaccine Stance Detection

**DOI:** 10.1007/s10916-026-02449-3

**Published:** 2026-08-08

**Authors:** Andreas Praschk, Valentin Fischill-Neudeck, Thomas Caspari, Hans-Peter Wiesinger

**Affiliations:** 1 Master Programme Public Health, Center for Public Health and Healthcare Research, Paracelsus Medical Private University, Strubergasse 21, Salzburg, 5020 Austria; 2 Institute of Nursing Science and Practice, Center for Public Health and Healthcare Research, Paracelsus Medical Private University, Strubergasse 21, Salzburg, 5020 Austria; 3 Faculty of Medicine, Paracelsus Medical Private University, Strubergasse 21, Salzburg, 5020 Austria; 4 Institute of General Practice, Family Medicine and Preventive Medicine, Center for Public Health and Healthcare Research, Paracelsus Medical Private University, Strubergasse 21, Salzburg, 5020 Austria

**Keywords:** Large language models, Chain-of-thought, Stance detection, Explainability, Vaccine hesitancy, Mixed-methods study

## Abstract

This mixed-methods study assessed whether reasoning-enabled large language models (LLMs) can classify stances towards COVID-19 vaccination on X (formerly Twitter) and whether model-generated chain-of-thought (CoT) summaries contain reasoning failures relevant to transparent and auditable public health applications. Zero-shot stance classification by o4-mini and Gemini 2.5 Flash (Gemini) was evaluated on 3,060 rehydrated COVID-19 vaccination tweets against human-annotated labels (positive, negative, neutral). We reported accuracy and macro-F1, measured CoT availability, and qualitatively analysed dual-error cases (tweets misclassified by both models) using Mayring’s content analysis guided by the FUTURE-AI framework. At each model’s best-performing setting, both models reached macro-F1 around 0.8, with o4-mini outperforming Gemini (accuracy 0.819 vs. 0.799, McNemar *p* = 0.0015; Δmacro-F1 = 0.020, 95% CI 0.008–0.032). Under the reasoning-intensive settings, CoT availability differed: Gemini returned a reasoning summary for all tweets, whereas o4-mini did so for 64.7%. Among 1,981 tweets with CoTs from both models, 295 (14.9%) were dual-errors; in 88.8%, both models produced the same wrong label, suggesting shared failure modes. Qualitatively, both models showed the same errors: target confusion (policy vs. vaccine), literal readings of sarcasm, and label–rationale mismatches, recurring across models despite their markedly different CoT lengths. Reasoning LLMs can therefore classify stance accurately, but their readiness for transparent public health applications depends on whether a CoT is available at all and whether it is coherent with the label it accompanies (label–rationale coherence). CoT availability, label–rationale coherence, and safeguards against systematic reasoning failures offer candidate explainability-readiness metrics, alongside accuracy, for trustworthy digital epidemiology.

**Supplementary Information:**

The online version contains supplementary material available at 10.1007/s10916-026-02449-3.

## Introduction

Social media is now an important channel both for public health communication and for the spread of vaccine misinformation. During the COVID-19 pandemic, such misinformation reduced the share of respondents in the UK and US who would definitely accept a vaccine by an estimated 6.2–6.4% points [1]. The same platforms that amplify polarising content through bots and trolls [3] are also the main data source for machine learning sentiment and stance analysis [2]. This tension motivates digital epidemiology methods that monitor evolving health narratives in near real time to support public health decisions [4]. Transformer-based language models [5, 6] are widely used for health-related stance detection [7, 11]. However, their accuracy can drift as online discourse changes, requiring regular and costly retraining on recent data [7]; and even when they perform well, they often remain opaque, which limits interpretability and auditability in high-stakes public health applications [8, 9]. Against this backdrop, stance detection itself has a longer methodological history. Stance detection was formalised as a benchmark task by Mohammad et al. [10], establishing evaluation protocols that remain widely used. ALDayel and Magdy [11] traced the field’s evolution from feature-engineered classifiers to fine-tuned transformer models such as BERT [6] and domain-specific variants [7]. Their survey showed that stance and sentiment are largely orthogonal constructs: in benchmark stance data, only about 35% of tweets expressing a favourable stance also carried positive sentiment, which illustrates why dedicated stance models, rather than sentiment proxies, are needed. These supervised approaches achieve strong results on fixed corpora but require labelled training data for each new target and provide limited insight into why a particular label was chosen. More recently, large language model (LLM)-based multi-agent frameworks have shown that zero-shot stance detection without fine-tuning is feasible [12], yet evaluation typically prioritises classification accuracy over reasoning quality. In health-related natural language processing (NLP), LLMs are increasingly applied to social media classification tasks. He et al. [13] reported prompt-dependent performance for GPT-3.5-Turbo on vaccine and mask-wearing tweet corpora, while Guo et al. [14] found that GPT-4 zero-shot classifiers outperformed SVM baselines in five of six health text tasks but still lagged behind fine-tuned models. Espinosa and Salathé [15] demonstrated that LLMs offer a scalable approach to analysing public health discourse, applying them to vaccination stance on social media. Across these studies, evaluation centres on aggregate performance and the quality and coherence of the models’ reasoning traces are hardly examined even where explanations are prompted [38]. This leaves a deployment-relevant gap: such studies show that LLMs can assign stance labels with acceptable accuracy, but not whether the reasoning artefacts available to human reviewers are consistently present and coherent with the assigned labels. This distinction matters in public health surveillance, where a fluent but incoherent explanation can lend credibility to an incorrect classification. A second shift is in how these models are deployed: through in-context learning, they adapt to new tasks from prompts alone, without retraining [16]. Reasoning-enabled LLMs such as o4-mini and Gemini 2.5 Flash [18, 19], the two models we evaluate here, go further: rather than requiring explicit step-by-step prompting to elicit reasoning [17], they generate a chain of thought (CoT) natively through model-internal reasoning and return a summary of it, exposed via API-level reasoning controls, alongside the label. Such reasoning traces have been proposed to make decisions more transparent and auditable. However, Turpin et al. [20] showed that a CoT can misrepresent a model’s actual decision process, producing plausible but unfaithful rationalisations; the faithfulness of post-hoc explanation tools such as LIME and SHAP [21, 22] has likewise been questioned [8]. Explainability in LLMs has been studied broadly [23], including in healthcare and medical applications [26], and CoT approaches have been applied to stance detection [24, 25], but the quality of the reasoning itself has rarely been audited at the case level. The requirement that a model’s explanation faithfully reflect its underlying decision process is a long-established objective in NLP: surveys review well over a hundred explanation methods through the lens of faithfulness [39], and a foundational distinction separates faithfulness (whether an explanation reflects the model’s actual reasoning) from plausibility (whether it merely appears convincing) [40]. Prior work has treated the association between a label and its rationale as a measurable, necessary property of faithful explanation [41] and shown that natural-language explanations and chain-of-thought traces frequently lack it, being unfaithful to the computation that produced the label [42, 43, 44]. Our explainability-readiness indicators therefore sit within this body of work, not apart from it. We therefore examine both classification performance and reasoning quality. Using an explanatory sequential design, we first measure performance and CoT availability, then qualitatively audit the cases that both models misclassify, assessing whether each CoT is coherent with the label it produced, to derive practical audit criteria for public health use. We make three contributions: we evaluate the zero-shot stance-classification performance of two reasoning-enabled LLMs on a manually annotated COVID-19 vaccine corpus from X; we operationalise two practical “explainability-readiness” indicators, CoT availability (measured directly) and label–rationale coherence (assessed qualitatively as the self-consistency between a model’s rationale and its label); and we develop an error taxonomy, grounded in the FUTURE-AI framework [27] and Mayring’s qualitative content analysis [28], to characterise systematic CoT failures relevant to public health.

## Methods

### Study Design

We used an explanatory sequential mixed-methods design [29]. In the quantitative phase, the reasoning-enabled LLMs o4-mini and Gemini were evaluated in zero-shot stance classification. We measured accuracy, macro-F1, and CoT availability. Quantitative results, including inconsistent CoT generation and recurring misclassifications, informed purposeful sampling for the qualitative phase. The qualitative audit analysed dual-error cases (tweets misclassified by both models) to identify common reasoning failures and evaluate label–rationale coherence. Following the logic of explanatory sequential designs [29], we deliberately limited the scope to two reasoning-enabled LLMs and a single manually annotated corpus. This focus enabled labour-intensive qualitative coding of 295 dual-error case bundles, which would not have been feasible with additional models or datasets.

### Dataset

We used the publicly accessible tweet-ID corpus from Cotfas et al. [30], in which three independent evaluators labelled each tweet and a majority vote determined the final stance. While this approach provides a reproducible benchmark, research on crowdsourced annotation suggests that majority-vote schemes may mask underlying label uncertainty [31]. The evaluation set comprised 4,341 tweet IDs collected between November 2020 and January 2021. Using twarc2 and the official X application programming interface (API), 3,060 tweets (70.5%) were successfully rehydrated (i.e., retrieved from the live X API by tweet ID); losses were due to deletions or changes of privacy settings. The final corpus included 1,123 neutral (36.7%), 1,017 positive (33.2%), and 920 negative tweets (30.1%).

### Inference Settings

LLMs were run in zero-shot mode using an identical system prompt (see Online Resource 1) without supervised fine-tuning. We evaluated o4-mini [18] at three reasoning-effort settings (low, medium, high) and Gemini 2.5 Flash [19] at three thinking budgets (150, 200, 250) (Table 1), computing accuracy and macro-F1 for each configuration on all 3,060 tweets (Table 2). Two of these configurations then served distinct roles. For the performance comparison we used each model’s best-performing setting: o4-mini at low effort and Gemini at a budget of 250. For the qualitative audit we deliberately used the most reasoning-intensive setting, o4-mini at high effort and Gemini at a budget of 250, because an audit needs as much analysable reasoning as possible: high effort returned a CoT for more tweets than low effort (64.7% vs. 57.8% for o4-mini), at the cost of a slightly lower accuracy (0.810 vs. 0.819; Table 2). Inference was performed against the vendor APIs using fixed model snapshots (Table 1). OpenAI o4-mini was accessed through the Responses API as model o4-mini-2025-04-16 with reasoning.summary = detailed; for o-series reasoning models, temperature and top_p are not user-configurable and the default internal sampling regime is used. Gemini 2.5 Flash was accessed through the Google GenAI software development kit (SDK) as model gemini-2.5-flash-preview-05-20 with no temperature, top-p, or seed override (SDK defaults). Concurrency, retry policy, the prompt checksum, and library versions are documented in Online Resource 1. All configurations were run once per model (a single pass, no row re-queried). The runs whose chain-of-thought outputs we audited were o4-mini at high effort (26 May 2025) and Gemini at a budget of 250 (5 June 2025).

**Table 1:** Model card inference configuration for both reasoning LLMs

Model	o4-mini	Gemini 2.5 Flash
Vendor	OpenAI	Google
Model snapshot	o4-mini-2025-04-16	gemini-2.5-flash-preview-05-20
Access method	Responses API (SDK openai 1.77.0)	GenAI API (SDK google-genai 1.17.0)
Reasoning configuration	reasoning.effort swept over {low, medium, high}; performance setting low, audit setting high; reasoning.summary = detailed	thinking_budget swept over {150, 200, 250}; performance and audit setting 250; include_thoughts = True
Sampling parameters	Not user-configurable for o-series models	SDK defaults (no temperature, top-p, or seed set)
Candidates per call	1	1
Concurrency	8 workers	8 workers
Retry policy	≤ 3 retries; 10 s base; 1.5× backoff	≤ 3 SDK retries + ≤ 5 worker retries; 10 s base; 1.5× backoff
Main inference date	26 May 2025	5 June 2025
System prompt	Identical across models	Identical across models
Output schema	Single label token + reasoning_summary	Single label token + extracted thought parts
Runtime environment	Python 3.13.3	Python 3.13.3

**Table 2:** Overall performance by model and setting (*N* = 3,060; 3 classes)

Model (setting)	Macro-*P*	Macro-*R*	Macro-F1 [95% CI]	Accuracy [95% CI]
o4-mini (Low)	0.822	0.826	0.820 [0.806–0.833]	0.819 [0.806–0.833]
o4-mini (Medium)	0.819	0.823	0.818 [0.804–0.831]	0.816 [0.803–0.830]
o4-mini (High)	0.816	0.818	0.811 [0.797–0.824]	0.810 [0.796–0.824]
Gemini-2.5-Flash (150)	0.789	0.794	0.786 [0.771–0.800]	0.786 [0.772–0.801]
Gemini-2.5-Flash (200)	0.795	0.800	0.792 [0.777–0.806]	0.792 [0.778–0.806]
Gemini-2.5-Flash (250)	0.800	0.806	0.800 [0.785–0.814]	0.799 [0.786–0.813]

Notes: Metrics are macro-averages (unweighted) across the three classes; Values in brackets are 95% bootstrap CIs (10,000 resamples). For each model, the row with the highest macro-F1 is bold. Macro-P and Macro-R denote macro-averaged precision and recall

### Outcome Measures

Throughout this study, ‘CoT’ refers to the reasoning summary returned by the vendor API, not the model’s internal reasoning process. CoT availability was defined as the proportion of inferences for which the API returned a non-missing, non-placeholder reasoning summary. Label–rationale coherence was defined as the agreement between the stance implied by a model’s CoT and the label it emitted. We summarised CoT availability descriptively (see Results), whereas label–rationale coherence was assessed qualitatively within the dual-error subset and captured through the traceability category of our coding scheme, whose T2 subcode (“inconsistent explanation”) marks cases in which the stated rationale and the emitted label diverge (see Qualitative analysis). The two reasoning artefacts are not directly comparable. For o4-mini, the Responses API returns a vendor-generated summary of the model’s hidden reasoning (reasoning.summary = detailed), whereas for Gemini the API returns a thought summary produced under a fixed 250-token thinking budget. In both cases the output is a vendor-mediated summary rather than a verbatim reasoning trace, so differences in CoT availability and length may partly reflect these API design and vendor reporting choices rather than model behaviour as such.

### Quantitative Analysis

Stance-level differences between o4-mini and Gemini on paired tweets (Δaccuracy) were tested using McNemar’s exact two-sided test on discordant pairs. Differences in macro-F1 (Δmacro-F1) were assessed with a paired percentile bootstrap (B = 10,000; seed = 42), providing mean differences, 95% confidence intervals, and two-sided bootstrap p-values. CoT availability was treated as a binary variable (reasoning summary present vs. missing/placeholder) for statistical analysis. Per-model accuracy and macro-F1, along with the proportion of dual-error cases where both models selected the same incorrect label, were summarised with 95% bootstrap confidence intervals (B = 10,000; seed = 42). The fixed seed (42) governs the bootstrap resampling of tweets, not the models’ decoding, so run-to-run decoding variability is not captured (see Limitations). Because each model’s headline figure is the best of three configurations, selected on the same tweets without correction for multiple comparisons, we treat the between-model comparison as exploratory rather than confirmatory. Quantitative analyses were conducted in Python (pandas, scikit-learn). Batched inference employed the official OpenAI and Google Generative AI SDKs.

### Qualitative Analysis

Because reasoning summaries can be fluent yet unfaithful [8, 20], we conducted an instance-level qualitative audit to identify systematic reasoning failures. For this purpose, the reasoning-intensive settings (see Inference settings) were applied across all 3,060 tweets. This led to 1,981 instances with CoTs from both models. From this set, we analysed all 295 dual-error cases. The unit of analysis was a case bundle: the source tweet text, its human-annotated reference label, each model’s predicted label, and each model’s reasoning summary. We developed the codebook using Mayring’s qualitative content analysis [28], employing a deductive–inductive approach, and refined it iteratively to achieve stability. The FUTURE-AI framework [27] is an international consensus guideline for trustworthy artificial intelligence (AI) in healthcare, defining six principles: fairness, universality, traceability, usability, robustness, and explainability. Because fairness and universality concern demographic equity and generalisability rather than reasoning quality, we aligned our main deductive categories with the remaining four: (i) traceability, (ii) usability, (iii) robustness, and (iv) explainability. Additionally, we defined a general category to capture recurring stylistic patterns in the CoTs that did not cleanly fit into the FUTURE-AI dimensions. Inductively, subcategories were derived through close reading of individual tweets and their associated CoTs. We inductively coded recurring themes in reasoning failures and grouped them under the main deductive categories to construct a coherent overall category system. The first author conducted the primary coding, discussing new and ambiguous codes with co-authors during regular sessions and resolving disagreements through consensus. To assess inter-coder reliability, the dual-error subset was re-coded by a second coder, who had taken part in the consensus discussions during codebook development and was therefore familiar with the coding rules, but had no access to the primary coding assignments. The second coding was performed on a coding-free copy of the data [50] against the frozen codebook (Online Resource 2), with the analysis rules fixed before coding began. Agreement was computed at the case level for each subcode and summarised as observed agreement and Cohen’s κ, with the prevalence- and bias-adjusted kappa (PABAK) [47] for codes with skewed distributions. Dimension-level estimates are reported in the Results; results for all subcodes are provided in Online Resource 3 (Table 1). Data management and coding were conducted in MAXQDA.

During the preparation of this work, the authors used DeepL for translating draft passages from German to English, Grammarly for improving readability and style, and an AI-assisted code completion tool (Cursor) for accelerating the writing of Python scripts in the data analysis pipeline. The authors reviewed and edited all outputs and take full responsibility for the content of the publication.

## Results

### Quantitative Performance and CoT Availability

o4-mini performed best at reasoning.effort = low (macro-F1 = 0.820; accuracy = 0.819), and Gemini at thinking_budget = 250 (macro-F1 = 0.800; accuracy = 0.799) (Table 2). Across the three classes, neutral was the hardest for both models (recall 0.71), whereas negative was recalled most reliably (0.91 for o4-mini at low effort, 0.93 for Gemini); precision was lowest for positive under o4-mini (0.78) and for negative under Gemini (0.79) (Table 2; per-class figures for all configurations are in Online Resource 4). Errors were not symmetric: neutral tweets were pushed towards a polarity rather than confused with each other. At high reasoning effort, o4-mini labelled 247 of the 1,123 neutral tweets positive and 115 negative; Gemini showed the same pattern (178 positive, 143 negative). On the paired set (*N* = 3,060), o4-mini outperformed Gemini: Δaccuracy = 0.020 (McNemar *p* = 0.0015) and Δmacro-F1 = 0.020 [95% CI 0.008–0.032], *p* = 0.0014 (paired bootstrap) (Table 3; Online Resource 5). Under the reasoning-intensive settings, CoT availability (as defined in Methods) was 100% for Gemini (3,060/3,060), and 64.7% for o4-mini (1,981/3,060). Median CoT length was 1,041 characters for Gemini and 457 characters for o4-mini (Tables 4 and 5; Online Resource 6). Restricting both models to the 1,981 tweets for which each produced a CoT, the median length was 457 characters for o4-mini and 1,048 for Gemini; Gemini’s median barely moves from its all-tweet value, so the two-fold difference is not an artefact of the tweets on which o4-mini returned no CoT and, as noted in the Methods, should be read in light of the different reasoning artefacts the two APIs return. Among the 1,981 tweets for which both APIs returned a reasoning summary, label agreement was 84.5% (1,674/1,981). Within this subset, 295 tweets were misclassified by both models. In 262 of these dual-error cases, the models assigned the same (incorrect) stance label, corresponding to a match proportion of 88.8% (262/295; 95% bootstrap CI 85.1–92.2). These shared errors were dominated by neutral tweets: 159 of the 262 (61%) were classed as positive (102) or negative (57); the remainder were mostly positive tweets softened to neutral (40) or flipped to negative (31). This shared-error pattern motivated the subsequent qualitative analysis (Table 6; Online Resource 7).

**Table 3:** Per-class precision, recall and F1 at the primary configurations (*N* = 3,060; 3 classes)

Model (setting)	Class	Precision	Recall	F1	Support
o4-mini (low)	negative	0.826	0.908	0.865	920
	neutral	0.860	0.712	0.779	1,123
	positive	0.779	0.857	0.816	1,017
Gemini 2.5 Flash (250)	negative	0.785	0.929	0.851	920
	neutral	0.815	0.714	0.761	1,123
	positive	0.799	0.776	0.787	1,017

Notes: Per-class metrics are shown for the primary configuration of each model (o4-mini at low reasoning effort; Gemini 2.5 Flash at thinking budget 250). Support is the number of tweets per class (*N* = 3,060)

**Table 4:**
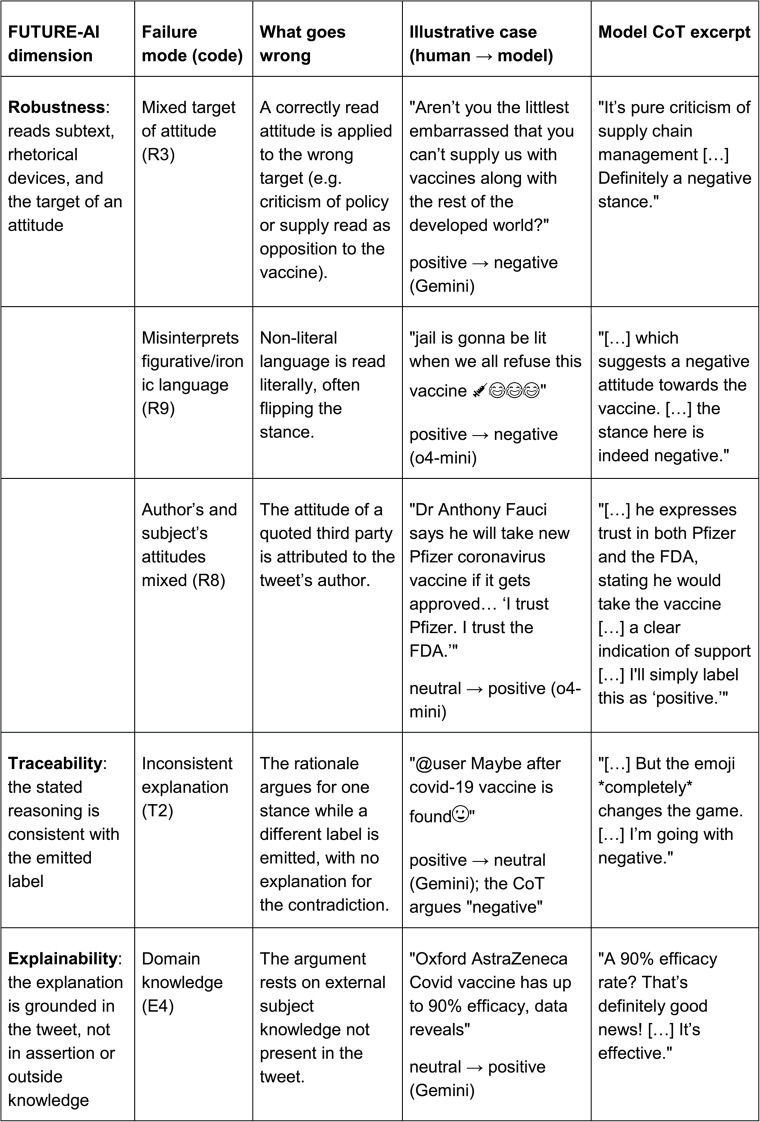
Recurring chain-of-thought failure modes in dual-error cases, by FUTURE-AI dimension

Notes: Dimension glosses describe what each categorycaptures; full definitions and a second anchor example per subcode are in thecodebook (Online Resource 2). Examples were selected to illustrate each failuremode as clearly as possible with a single case, not by frequency of occurrence;the tweets shown are publicly available posts reproduced verbatim forillustration, with account handles retained only where the addressee is apublic institution or official. Only tweet IDs are redistributed (OnlineResource 8)

### Qualitative Analysis of Failures in Model-Generated Reasoning Summaries

**Fig. 1:**
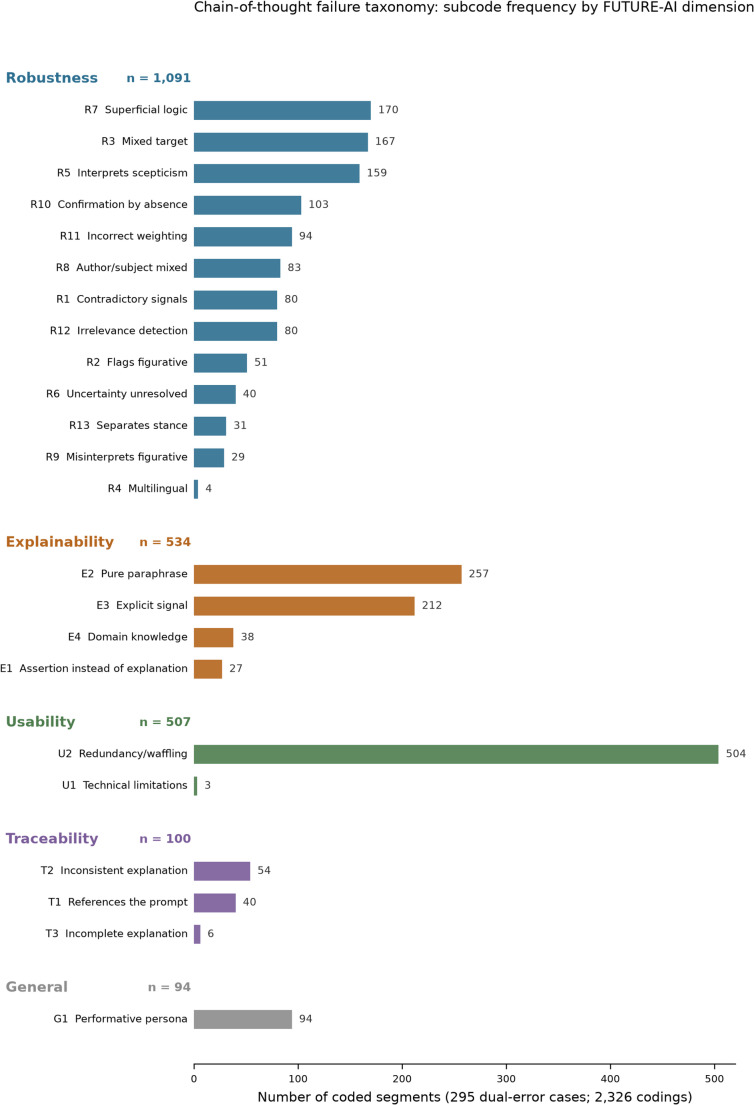
Chain-of-thought failure taxonomy: coding frequency per inductive subcode, grouped by FUTURE-AI dimension and ordered by frequency (n = 2,326 coded segments across 295 dual-error cases) Notes: Segments are coded text spans within the models’ reasoning summaries; a single case bundle can carry multiple subcodes, so counts sum to more than 295.

Guided by the FUTURE-AI framework, the final coding system comprised five deductive categories and 23 inductive subcategories; Fig. 1 shows the full taxonomy with the frequency of each subcode. Across the 23 subcodes applied at the case level (6,785 paired decisions), observed agreement was 81.4% and Cohen’s κ was 0.44, moderate by the conventions of Landis and Koch [48]. Per dimension, observed agreement ranged from 57.3% (Explainability) to 94.2% (Robustness); because both coders assigned Usability and Robustness codes in nearly every case, κ is uninformative for these dimensions despite agreement above 93% (PABAK 0.86 and 0.88 [47]). We focus on the three categories that concern returned reasoning summaries (Robustness, Traceability, and Explainability); Usability and General capture stylistic rather than reasoning-level patterns and are reported in the full codebook (Online Resource 2). Table 4 gives representative failure modes for each, with an illustrative case and the corresponding reasoning summary.

## Discussion

This mixed-methods audit shows that strong zero-shot accuracy (macro-F1 ≈ 0.8) coexists with systematic weaknesses in the models’ reasoning summaries that directly affect auditability. The two models differ sharply in whether a CoT is available at all (Gemini for every tweet, o4-mini for about two-thirds); and where both did produce one, they tended to fail together, since 88.8% of the 295 dual-error cases carried the same incorrect label. This points to shared rather than idiosyncratic failure modes. Both models perform at the upper end of reported zero-shot results on health-related social media stance tasks. For comparison, He et al. report GPT-3.5-Turbo weighted F1 of 0.55 on HPV-vaccine tweets using a generic prompt, and up to 0.85 on mask-wearing tweets using a data-specific prompt [13], while GPT-4 zero-shot classifiers can still lag behind supervised baselines by roughly 0.15 F1 on average across health-related social media classification tasks [14]. Very high accuracies reported for GPT-4 on heavily neutral-dominated vaccine stance corpora largely reflect the strength of the majority-class baseline rather than robust three-way discrimination, with 74.1–77.3% of expert labels and 89.5–98.1% of crowd labels being neutral; an always-neutral classifier already attains 0.74–0.98 accuracy [15]. Although the between-model differences reached statistical significance, their magnitude was small (Δaccuracy = 0.020; Δmacro-F1 = 0.020), so we do not regard either model as practically superior for stance classification; the more consequential differences lie in CoT availability and coherence rather than in headline accuracy. Beyond CoT availability, o4-mini produced shorter CoT explanations than Gemini. The models’ failures cluster into three audit problems that build on one another: rationales that do not support the label they accompany, reasoning that addresses the wrong target, and detailed, seemingly nuanced language that can make either failure look reliable. This was most evident in the decoupling of reasoning from judgement (T2), where models provided detailed analysis but issued a final label that contradicted their own rationale. They also faced difficulties with contextual disentanglement, often conflating criticism of political processes with opposition to the vaccine (R3, R8) and failing to interpret non-literal language such as sarcasm (R9). This pattern is consistent with prior stance-detection work showing that CoT prompting can suffer from implicit stance confusion and stance-label hallucination, and that LLM-generated stance labels used in CoT-based pipelines may contain errors requiring mitigation strategies [24, 25]. Both models exhibited failures in every category, so the taxonomy is not an artefact of one model: 40% of the coded segments came from o4-mini and 60% from Gemini, an imbalance largely explained by Gemini’s reasoning summaries being roughly twice as long; the one clear exception is the General category (performative persona), which was almost exclusively Gemini (92 vs. 2 segments). That o4-mini’s accuracy fell slightly as reasoning effort increased (0.819 to 0.810) is consistent with the qualitative pattern we observed, in which additional reasoning was often spent over-reading factual content as implicit endorsement; on o4-mini’s own effort-loss cases this reasoning most frequently carried the R7 (superficial logic) and U2 (redundancy and waffling) subcodes, rather than improving the final judgement. More fundamentally, the chain-of-thought traces analysed here are vendor-provided reasoning artefacts, not verified records of the models’ internal computation. Controlled audits of reasoning-tuned models show that they verbalise the hint they actually rely on in only a minority of cases (overall faithfulness of 25% and 39% for two reasoning models), with faithfulness declining further on harder inputs [45], and consistency checks such as ours assess output-level self-consistency between the prediction and the stated rationale, not fidelity to the underlying decision process [40, 46]. Our ‘CoT failure modes’ should therefore be read as failures of the stated rationale, not as direct evidence about the models’ internal reasoning.

Strong aggregate metrics can hide problems that distort surveillance data. Misclassifying criticism of government policy as anti-vaccine sentiment or interpreting pro-vaccine sarcasm literally can inflate or underestimate estimates of vaccine hesitancy, potentially misguiding public health interventions. Inconsistent CoTs further complicate human oversight, as plausible local explanations can be misleading and encourage over-reliance even when decisions are wrong [8, 32], a concern underscored by evidence that CoT traces can be systematically unfaithful to a model’s actual reasoning process [20, 44]. Although our study focused on COVID-19 vaccine discourse, the core question is not COVID-specific: whether LLM reasoning faithfully reflects how the model actually decides. This question applies to any public health setting where LLMs interpret social media, including outbreak misinformation surveillance, behavioural risk factor monitoring, and stance assessment for other vaccines or health policies. The failure modes described here can guide similar audits. Accuracy-only benchmarks are therefore insufficient for evaluating LLMs in public health applications. CoT availability and label–rationale coherence reveal errors invisible to aggregate performance measures. This reflects a broader shift in health AI evaluation, where predictive performance alone is increasingly regarded as insufficient for systems that inform clinical or public health decisions, and the quality of a model’s explanation is treated as a first-class evaluation target [23, 27]. Embedding these properties within governance frameworks such as AI4People’s “Good AI Society” [33] and FUTURE-AI’s principles [27], and framing them in “Model Facts”-style disclosures, can enhance transparency and support context-appropriate use, ensuring that LLM outputs are treated as inputs for further investigation rather than definitive epidemiological endpoints [34, 35]. Read this way, CoT availability and label–rationale coherence act as deployment-readiness checks: before a reasoning LLM is trusted in a surveillance pipeline, one can ask whether it reliably produces a rationale at all, and whether that rationale is consistent with the label it assigns.

Several limitations qualify these findings. The 29.5% attrition rate from rehydrating the corpus may introduce selection bias and limit generalisability to later discourse, other platforms, or additional languages. We used the stance labels from Cotfas et al. [30] as our benchmark, noting that they were manually annotated rather than formally expert-adjudicated. The original study does not report inter-annotator agreement or annotator qualifications; residual label noise at the neutral/negative boundary may therefore have inflated the dual-error rate for ambiguous posts. Methodologically, our evaluation is limited to two LLMs tested zero-shot with fixed prompts, so different models or prompting strategies (e.g., few-shot) could yield different results. Analysing only the dual-error cases supports interpreting the observed failure patterns as model-agnostic, but prevents estimating the prevalence of each failure type in the full prediction set or among cases without CoT. Because the codebook was developed on and calibrated to misclassified cases, extending it to correctly classified tweets would require re-validation; whether the patterns identified here also occur when the models are right therefore remains an open question for future work. Pooled inter-coder agreement was moderate (κ = 0.44), in line with published agreement levels for human judgements of model reasoning quality [49]. Disagreement concentrated in the descriptive Explainability codes (pure paraphrase, explicit signal), which the first coder applied more liberally than the second; agreement was higher for the interpretive failure codes. Although inter-coder reliability was assessed using the final codebook, the second coder contributed to its development. Accordingly, the reported agreement reflects a within-team assessment rather than independent external validation, and the taxonomy should be considered exploratory until validated by independent coders and additional datasets. A further limitation concerns run-to-run variability. All estimates derive from a single inference run per configuration. o4-mini exposes no user-controllable sampling parameters (temperature and top-p are rejected), and although Gemini exposes a seed field, neither API guarantees deterministic decoding, so we could not bound run-to-run variation directly. Decoding variability could therefore shift the two-point accuracy difference, the CoT-availability percentages, and the exact composition of the 295 dual-error cases; the bootstrap confidence intervals capture uncertainty from tweet sampling only. Finally, the rapid evolution of commercial LLMs means that model behaviour and CoT functionality may shift over time, limiting temporal generalisability.

## Conclusions

In this audit, two reasoning-enabled LLMs classified COVID-19 vaccine stance accurately in zero-shot use, yet both displayed the same recurring CoT failures (target confusion, sarcasm misinterpretation, and label–rationale incoherence). For public health applications, evaluation should therefore extend beyond accuracy to explainability readiness (CoT availability and label–rationale coherence), and LLM outputs should serve as inputs for human review rather than as endpoints in themselves. Future work should test whether these patterns hold across other reasoning-enabled LLMs and configurations, apply the codebook to a sample of correctly classified cases to establish whether the identified patterns are specific to misclassification or reflect properties of model-generated reasoning more generally, and extend the audit to further public health topics and languages, including low-resource settings [36]. It should also incorporate the remaining FUTURE-AI dimensions (fairness and universality) and evaluate targeted mitigations, such as sarcasm detection and rule-based label–rationale checks.

## Supplementary Information

Below is the link to the electronic supplementary material.

Supplementary Material 1

Supplementary Material 2

Supplementary Material 3

Supplementary Material 4

Supplementary Material 5

Supplementary Material 6

Supplementary Material 7

Supplementary Material 8

Supplementary Material 9

## Data Availability

For all 3,060 tweets, Online Resource 7 provides the reference label, each model’s prediction, CoT availability, dual-error status, and the assigned failure-mode subcodes, keyed by tweet ID. In line with X’s Developer Policy, we do not redistribute tweet text.
