# Supplementary material for: Beyond Accuracy: A Mixed-Methods Audit of Chain-of-Thought Failures in LLM-Based COVID-19 Vaccine Stance Detection
Source: J Med Syst. 2026 Aug 8;50(1):123. doi: 10.1007/s10916-026-02449-3 (PMC13452790; doi:10.1007/s10916-026-02449-3)
Supplement: Supplementary file 1 [file 10916_2026_2449_MOESM1_ESM.docx]

**Supplementary material for:**
Beyond accuracy: a mixed-methods audit of chain-of-thought failures in LLM-based COVID-19 vaccine stance detection.

**Journal:** Journal of Medical Systems

Andreas Praschk, BSc; Valentin Fischill-Neudeck, PhD, MScN; Thomas Caspari, Dr rer nat; Hans-Peter Wiesinger, Dr rer nat, LLB oec

Paracelsus Medical Private University, Center for Public Health and Healthcare Research, Strubergasse 21, 5020 Salzburg, Austria

Corresponding author: Hans-Peter Wiesinger, E-mail: hans-peter.wiesinger@pmu.ac.at

**Table 1** Inter-coder agreement for the 23 subcodes applied at the case level (N = 295 dual-error cases)

| **Subcode** | **Coder 1 (n)** | **Coder 2 (n)** | **a** | **b** | **c** | **d** | **Observed agreement (%)** | **Positive agreement** | **Cohen’s κ** | **PABAK** |
| --- | --- | --- | --- | --- | --- | --- | --- | --- | --- | --- |
| T1 | 36 | 11 | 7 | 29 | 4 | 255 | 88.8 | 0.298 | 0.255 | 0.776 |
| T2 | 49 | 21 | 16 | 33 | 5 | 241 | 87.1 | 0.457 | 0.397 | 0.742 |
| T3 | 6 | 6 | 1 | 5 | 5 | 284 | 96.6 | 0.167 | 0.149 | 0.932 |
| U1* | 3 | 1 | 1 | 2 | 0 | 292 | 99.3 | 0.500 | 0.497 | 0.986 |
| U2 | 295 | 275 | 275 | 20 | 0 | 0 | 93.2 | 0.965 | 0.000 | 0.864 |
| R1 | 64 | 70 | 38 | 26 | 32 | 199 | 80.3 | 0.567 | 0.440 | 0.607 |
| R2 | 39 | 22 | 13 | 26 | 9 | 247 | 88.1 | 0.426 | 0.366 | 0.763 |
| R3 | 99 | 92 | 51 | 48 | 41 | 155 | 69.8 | 0.534 | 0.311 | 0.397 |
| R4* | 2 | 2 | 2 | 0 | 0 | 293 | 100.0 | 1.000 | 1.000 | 1.000 |
| R5 | 114 | 108 | 58 | 56 | 50 | 131 | 64.1 | 0.523 | 0.235 | 0.281 |
| R6 | 37 | 8 | 4 | 33 | 4 | 254 | 87.5 | 0.178 | 0.139 | 0.749 |
| R7 | 106 | 39 | 23 | 83 | 16 | 173 | 66.4 | 0.317 | 0.154 | 0.329 |
| R8 | 48 | 28 | 10 | 38 | 18 | 229 | 81.0 | 0.263 | 0.163 | 0.620 |
| R9 | 21 | 30 | 6 | 15 | 24 | 250 | 86.8 | 0.235 | 0.165 | 0.736 |
| R10 | 81 | 26 | 19 | 62 | 7 | 207 | 76.6 | 0.355 | 0.256 | 0.532 |
| R11 | 67 | 32 | 12 | 55 | 20 | 208 | 74.6 | 0.242 | 0.112 | 0.492 |
| R12 | 64 | 63 | 34 | 30 | 29 | 202 | 80.0 | 0.535 | 0.408 | 0.600 |
| R13 | 27 | 9 | 4 | 23 | 5 | 263 | 90.5 | 0.222 | 0.185 | 0.810 |
| E1 | 24 | 15 | 2 | 22 | 13 | 258 | 88.1 | 0.103 | 0.043 | 0.763 |
| E2 | 199 | 87 | 58 | 141 | 29 | 67 | 42.4 | 0.406 | −0.008 | −0.153 |
| E3 | 183 | 78 | 62 | 121 | 16 | 96 | 53.6 | 0.475 | 0.166 | 0.071 |
| E4 | 37 | 19 | 13 | 24 | 6 | 252 | 89.8 | 0.464 | 0.414 | 0.797 |
| G1 | 91 | 58 | 56 | 35 | 2 | 202 | 87.5 | 0.752 | 0.673 | 0.749 |
| **All subcodes (pooled)** | **1692** | **1100** | **765** | **927** | **335** | **4758** | **81.4** | **0.548** | **0.437** | **0.628** |

*Notes: Unit of analysis = case × subcode (subcode assigned versus not assigned to a case). Coder 1 = first author (primary coding); Coder 2 = blinded second coder, who re-coded all 295 dual-error case bundles on a coding-free copy of the data against the frozen codebook (Online Resource 6). a = cases in which both coders assigned the subcode; b = Coder 1 only; c = Coder 2 only; d = neither coder. Observed agreement = (a + d)/295. Positive agreement = 2a/(2a + b + c). PABAK = prevalence- and bias-adjusted kappa = 2 × observed agreement − 1 (Byrt et al. 1993). Pooled values are computed over all 23 × 295 = 6,785 decisions; the pooled Krippendorff's α was 0.43. * Below the pre-specified floor of five positive cases; these subcodes are interpreted only within the pooled estimate.*
