# Supplementary material for: Beyond Accuracy: A Mixed-Methods Audit of Chain-of-Thought Failures in LLM-Based COVID-19 Vaccine Stance Detection
Source: J Med Syst. 2026 Aug 8;50(1):123. doi: 10.1007/s10916-026-02449-3 (PMC13452790; doi:10.1007/s10916-026-02449-3)
Supplement: Supplementary file 2 [file 10916_2026_2449_MOESM2_ESM.docx]

**Supplementary material for:**
Beyond accuracy: a mixed-methods audit of chain-of-thought failures in LLM-based COVID-19 vaccine stance detection.

**Journal:** Journal of Medical Systems

Andreas Praschk, BSc; Valentin Fischill-Neudeck, PhD, MScN; Thomas Caspari, Dr rer nat; Hans-Peter Wiesinger, Dr rer nat, LLB oec

Paracelsus Medical Private University, Center for Public Health and Healthcare Research, Strubergasse 21, 5020 Salzburg, Austria

Corresponding author: Hans-Peter Wiesinger, E-mail: hans-peter.wiesinger@pmu.ac.at

**Table** 4 CoT availability and length statistics by model configuration (N = 3,060)

| **Model (setting)** | **CoT availability (%)** | **CoT length, mean (chars)** | **CoT length, median (chars)** | **CoT length, p10–p90 (chars)** |
| --- | --- | --- | --- | --- |
| **o4-mini (Low)** | 57.84 | 471 | 472 | 414–525 |
| o4-mini (Medium) | 59.18 | 458 | 459 | 391–523 |
| o4-mini (High) | 64.74 | 458 | 457 | 389–533 |
| **Gemini-2.5-Flash (150)** | 100 | 902 | 878 | 669–1168 |
| Gemini-2.5-Flash (200) | 100 | 1029 | 1018 | 770–1298 |
| Gemini-2.5-Flash (250) | 100 | 1047 | 1041 | 761–1333 |

*Notes: CoT availability = share of rows with a non-missing, non-*"*<no summary>" reasoning_summary. Length statistics are computed only on available CoTs. Statistics restricted to the tweets with a CoT from both models are reported in the additional table below.*

**Table 5** CoT length statistics restricted to the N = 1,981 tweets with a CoT from both models (audit configurations)

| **Model (setting)** | **CoT length, mean (chars)** | **CoT length, median (chars)** | **CoT length, p10–p90 (chars)** |
| --- | --- | --- | --- |
| **o4-mini (High)** | 458 | 457 | 389–533 |
| Gemini-2.5-Flash (250) | 1053 | 1048 | 766–1343 |
