# Supplementary material for: Beyond Accuracy: A Mixed-Methods Audit of Chain-of-Thought Failures in LLM-Based COVID-19 Vaccine Stance Detection
Source: J Med Syst. 2026 Aug 8;50(1):123. doi: 10.1007/s10916-026-02449-3 (PMC13452790; doi:10.1007/s10916-026-02449-3)
Supplement: Supplementary file 3 [file 10916_2026_2449_MOESM3_ESM.docx]

**Supplementary material for:**
Beyond accuracy: a mixed-methods audit of chain-of-thought failures in LLM-based COVID-19 vaccine stance detection.

**Journal:** Journal of Medical Systems

Andreas Praschk, BSc; Valentin Fischill-Neudeck, PhD, MScN; Thomas Caspari, Dr rer nat; Hans-Peter Wiesinger, Dr rer nat, LLB oec

Paracelsus Medical Private University, Center for Public Health and Healthcare Research, Strubergasse 21, 5020 Salzburg, Austria

Corresponding author: Hans-Peter Wiesinger, E-mail: hans-peter.wiesinger@pmu.ac.at

**Table3** Paired comparison of best configurations

| **Comparison** | **n** | **ΔAccuracy** | **McNemar p** | **ΔMacro-F1 [95% CI]** | **p (bootstrap)** |
| --- | --- | --- | --- | --- | --- |
| **o4-mini (low) vs Gemini-2.5-Flash (250)** | 3,060 | 0.020 | 0.0015 | 0.020 [0.008–0.032] | 0.0014 |

*Note: ΔAccuracy is computed as (o4-mini − Gemini-2.5-Flash). Accuracy tested with McNemar*’*s exact test (two-sided); ΔMacro-F1 reported with percentile 95% bootstrap CIs (10,000 resamples) and two-sided bootstrap p. Accuracy = micro-F1.*
