# Supplementary material for: Beyond Accuracy: A Mixed-Methods Audit of Chain-of-Thought Failures in LLM-Based COVID-19 Vaccine Stance Detection
Source: J Med Syst. 2026 Aug 8;50(1):123. doi: 10.1007/s10916-026-02449-3 (PMC13452790; doi:10.1007/s10916-026-02449-3)
Supplement: Supplementary file 4 [file 10916_2026_2449_MOESM4_ESM.docx]

**Supplementary material for:**
Beyond accuracy: a mixed-methods audit of chain-of-thought failures in LLM-based COVID-19 vaccine stance detection.

**Journal:** Journal of Medical Systems

Andreas Praschk, BSc; Valentin Fischill-Neudeck, PhD, MScN; Thomas Caspari, Dr rer nat; Hans-Peter Wiesinger, Dr rer nat, LLB oec

Paracelsus Medical Private University, Center for Public Health and Healthcare Research, Strubergasse 21, 5020 Salzburg, Austria

Corresponding author: Hans-Peter Wiesinger, E-mail: hans-peter.wiesinger@pmu.ac.at

**Codebook final**

**Main Category T: TRACEABILITY
Definition:** This category assesses the traceability and logical consistency of the model’s reasoning. Errors here diminish trust in the explanation, even if parts of the analysis are correct. They include direct contradictions between reasoning and results, as well as incomplete or hallucinated justifications.
**Focus:** Is the reasoning complete, internally consistent, and free from hallucinated instructions?

**Subcategory T1: References the prompt
Definition**: The model actively justifies an analytical step or its final decision with a rule or definition from the system prompt. Includes both correct and hallucinated prompt references.
**Coding rule**: Code if the model explicitly refers to a (hallucinated) instruction from the user to support its label.
**Differentiation:** vs. G1 (role-playing/persona), where the model relies on its own internal expertise, while T1 relies on a system-provided prompt.

**Anchor Example - 1**
Tweet: "Petition: Prioritise teachers, school and childcare staff for Covid-19 vaccination https://t.co/pPNf5EMbAy" (Tweet ID: 1346717142020988928)
Labels: Human: neutral • Gemini: positive
CoT-Excerpt: "[…] Given this context, the tweet's sentiment clearly aligns with the "positive" definition: support, approval, a favourable outlook, or a positive experience."

**Anchor Example - 2**
Tweet: "Pfizer, BioNTech COVID-19 vaccine trials raise hopes for ‘early’ 2021 delivery #COVID19 #vaccine https://t.co/eva1le0YJ4 via @user" (Tweet ID: 1326206232890241025)
Labels: Human: neutral • o4-mini: positive
CoT-Excerpt: "[…] I remember that a positive stance means support or a favorable outlook, while a negative one indicates criticism, and neutral means there’s no clear stance. Therefore, I'll respond with the single word: positive."

**Subcategory T2: Inconsistent explanation
Definition:** The reasoning in the CoT logically leads to a specific conclusion (e.g., "neutral"), but the final label output by the model is different (e.g., "positive") without any explanation for this contradiction.
**Coding rule:** Code if the summary or conclusion of the CoT clearly contradicts the final label.
**Differentiation:** vs. R6 (uncertainty), where the model uses its indecision as a reason for its (often neutral) label, while in T2 the CoT clearly points in a direction that contradicts the final label.

**Anchor Example - 1**
Tweet: "@user Maybe after covid-19 vaccine is found🙄" (Tweet ID: 1326238765027905543)
Labels: Human: positive • Gemini: neutral
CoT-Excerpt: "[…] But the emoji *completely* changes the game. It’s hard to imagine that anyone is using that expression to convey excitement or support. I'm going with negative."

**Anchor Example - 2**
Tweet: "The standard coronavirus tests are throwing up a huge number of positive cases daily. These tests are done based on faulty WHO protocols which are designed to include false positives cases as well." #COVID19 @user #ikvaccineerniet <https://t.co/OloHKfC4St>" (Tweet ID: 1329509082626220033)
Labels: Human: neutral • o4-mini: positive
CoT-Excerpt: "[…] I conclude that the overall stance on vaccination is neutral since it doesn’t express a clear opinion."

**Subcategory T3: Incomplete explanation
Definition:** The CoT ends abruptly, often before the decisive sentence that justifies the classification. The CoT is thus incomplete and incomprehensible.
**Coding rule:** Code if the CoT is obviously incomplete and breaks off before the final conclusion.
**Differentiation:** vs. E1 (assertion instead of explanation), where the explanation is present but unfounded.

**Anchor Example - 1**
Tweet: "The Risks of the Rushed Vaccine Do Not Outweigh the Benefits▸ https://t.co/2NuhPIxvqc#Covid19 #Vaccine <https://t.co/HZrSmCfkye>" (Tweet ID: 1337761725194579968)
Labels: Human: negative • Gemini: positive
CoT-Excerpt: "[…] Therefore, my assessment is:"

**Anchor Example - 2**
Tweet: "Moderna and other COVID-19 vaccines candidates like the Pfizer - BioNTech SE, AstraZeneca and NOVAVAX INC. are changing the way vaccines are made with revolutionary and technological breakthroughs. Moderna at the start of the week…https://t.co/XzHJNTEBRy <https://t.co/ifdcIwZvvt>" (Tweet ID: 1329059922554671105)
Labels: Human: neutral • Gemini: positive
CoT-Excerpt: "[…] My single-word classification, therefore, would be:"

**Main Category U: USABILITY
Definition**: This category evaluates aspects of the model output that affect usability and comprehensibility. It includes both stylistic flaws such as redundancy and the acknowledgment of technical limitations that influence the analysis.
**Focus:** How helpful and understandable is the CoT for a human analyst?

**Subcategory U1: Mentions technical limitations
Definition:** The model cites a technical limitation on its part (e.g., the inability to follow links or analyse images) as the reason for uncertainty or an incomplete analysis. **Coding rule:** Code if the model explicitly refers to a technical limitation ("I need to read the link"). **Differentiation:** vs. R6 (Identifies uncertainty), where the uncertainty lies in the text itself, not in a technical limitation.

**Anchor Example - 1**
Tweet: "This is great news. But would you be in a rush to try a brand new vaccine? <https://t.co/YSb4ueMCVY>" (Tweet ID: 1325771002282975237)
Labels: Human: positive • Gemini: negative
CoT-Excerpt: "[…] It suggests that there's a counterpoint being made, and without the URL's context, I can't be sure what "great news" actually refers to. Is it the vaccine? Is it the possibility of a vaccine? I just don't have enough to work with here."

**Anchor Example - 2**
Tweet: "I need to know WHY. WHY would ANYONE with access to vaccines do this? <https://t.co/3lg2MFC1dB>" (Tweet ID: 1344657246412800000)
Labels: Human: neutral • Gemini: positive
CoT-Excerpt: "The key is to figure out what ‘this’ refers to in the context […] So I need to read the link in order to give a definitive answer."

**Subcategory U2: Redundancy and Waffling
Definition:** The CoT contains filler material, redundant phrases, repeats the task, or quotes the tweet verbatim without contributing new information or analysis steps.
**Coding rule:** Code if the CoT begins or ends with a task restatement + filler without adding textual evidence (e.g., "Okay, I need to classify this tweet...").
**Differentiation:** vs. E2 (pure paraphrase), which repeats the content of the tweet, not the task.

**Anchor Example - 1**
Tweet: "@user @user @user, "Transparency is needed for #Covid19 vaccine trials" STAT <https://t.co/CHYuYmiegK>" (Tweet ID: 1325672694151569408)
Labels: Human: negative • Gemini: neutral
CoT-Excerpt: "**My Analysis of the Tweet's Stance** Okay, so I'm being asked to classify the stance of this tweet regarding COVID-19 vaccination. It seems straightforward enough: positive, negative, or neutral."

**Anchor Example - 2**
Tweet: "@user @user The SIV and HIV example was merely a comparison. I think you may have heard about the Oxford vaccine. It is a genetically modified common cold virus that originally infected chimpanzees. #covid19 #Covid19Aus <https://t.co/47cAHaDK8f>" (Tweet ID: 1331084752942600197)
Labels: Human: negative • o4-mini: neutral
CoT-Excerpt: "**Classifying vaccine stance** The user wants to classify the stance toward COVID-19 vaccination in a tweet."

**Main Category R: ROBUSTNESS
Definition:** This category assesses the model's ability to maintain consistent performance when confronted with linguistic variation. Errors here emerge when the model encounters complex, ambiguous, or context-dependent phenomena, revealing fundamental weaknesses in interpreting subtext, rhetorical devices, and assigning attitudes correctly.
**Focus:** How well does the model handle complex, ambiguous, or difficult content?

**Subcategory R1: Contradictory signals weighed
Definition:** The model explicitly recognizes that a tweet contains contradictory or ambiguous signals (e.g., criticism of one aspect but praise for another; neutral reporting of a positive event). It identifies this conflict in its argumentation.
**Coding rule:** Code when the CoT explicitly points out a contradiction or ambiguity between different signals in the tweet.
**Differentiation:** vs. R11 (incorrect signal weighting), where the weighing itself is the core of the error.

**Anchor Example - 1**
Tweet: "Pfizer’s Covid-19 vaccine passed a critical process on Thursday when a panel of experts formally recommended that the Food and Drug Administration authorize the vaccine.**#covid19 #donttaketheirvaccines #science #biotechnology #pfizer <https://t.co/oTfkvcZt56>" (Tweet ID: 1337190713897967616)
Labels: Human: neutral • o4-mini: negative
CoT-Excerpt: "It seems like a conflict between the positive factual statement and the negative sentiment from the hashtags."

**Anchor Example - 2**

Tweet: "It’s good news that one dosage regimen give 90% efficacy while it’s a concern that the other dosage regimen is a low 62% awaiting results from #SII #SERUMINSTITUTE India on their India specific vaccine @user #COVID19India #vaccine #COVID19 <https://t.co/s13fdq8D28>" (Tweet ID: 1330779039468900352)
Labels: Human: positive • Gemini: neutral
CoT-Excerpt: "This isn't a simple ‘yay vaccine’ or ‘nay vaccine’ situation. It highlights that one regimen boasts impressive 90% efficacy – that's definitely a positive aspect. But then there's a flip side: another regimen shows a less impressive 62% efficacy, which understandably raises a concern."

**Subcategory R2: Flags Figurative/ironic language
Definition:** The model recognizes that a statement is not to be taken literally and identifies the rhetorical device used, such as sarcasm, irony, or a metaphor.
**Coding rule:** Code when the CoT explicitly names a form of figurative or ironic language.
**Differentiation:** vs. R9 (misinterprets figurative/ironic language), where the irony is not identified as such in the first place.

**Anchor Example - 1**
Tweet: "Great. Didn't wear a mask 1/2 the time, attended all the super spreader events, but he gets the vaccine. Typical <https://t.co/WJyJboOKwO>" (Tweet ID: 1339934020826787849)
Labels: Human: positive • Gemini: negative
CoT-Excerpt: "‘Great.’ immediately stands out, but given the rest of the sentence, it's clearly dripping with sarcasm. It sets the tone."

**Anchor Example - 2**
Tweet: ""wHy WuD I gEt a vAccIne FoR sOmeFiNk with sUch a HiGh sUrviVal RaTe?!" Great point. I mean, we only vaccinate against things that are highly likely to kill you....... FUCK MY LIFE #COVID19 #Covid19UK #coronavirusuk #coronavirus #coronavirusvaccine #Pfizer https://t.co/vDontzHw80" (Tweet ID: 1325927702424117249)
Labels: Human: negative • o4-mini: positive
CoT-Excerpt: "The user's tweet is expressing sarcasm about the belief that a high survival rate negates the need for vaccination against COVID-19."

**Subcategory R3: Mixed target of attitude
Definition:** The model correctly identifies an attitude but applies it to the wrong target. For example, criticism of the political process is misinterpreted as criticism of the vaccine itself.
**Coding rule:** Code when the attitude toward a related concept (e.g., vaccine mandate, rollout speed, transparency) is transferred to the vaccine.
**Differentiation:** vs. R8 (attitude of author and subject mixed), where it concerns different people, not different topics.

**Anchor Example - 1**
Tweet: "Would you go along with a Gov't mandate that you not only have to submit to a #COVID19 #vaccine, but you would also have to show proof you got the vaccine by putting a mark on your hand or forehead, and you couldn't buy or sell anything unless you did?Asking for a friend" (Tweet ID: 1340033999620726785)
Labels: Human: neutral • o4-mini: negative
CoT-Excerpt: "Since the tone implies a dystopian scenario and expresses opposition, I would classify this as a negative stance toward the idea of such mandates."

**Anchor Example - 2**
Tweet: "@user Aren't you the littlest embarrassed that you can't supply us with vaccines along with the rest of the developed world? <https://t.co/88LiASNm4C>" (Tweet ID: 1332411132573675520)
Labels: Human: positive • Gemini: negative
CoT-Excerpt: "It's pure criticism of supply chain management, and how Canada measures up in the global race for vaccines. Definitely a negative stance."

**Subcategory R4: Interprets multilingual content
Definition**: The model recognises non-English elements and attempts interpretation/translation.
**Coding rule:** Code if the model explicitly identifies a language and offers a translation.
**Differentiation:** vs. R9 (misinterprets figurative/ironic language), where the challenge lies in rhetoric rather than language barriers.

**Anchor Example - 1**
Tweet: "The standard coronavirus tests are throwing up a huge number of positive cases daily. These tests are done based on faulty WHO protocols which are designed to include false positives cases as well." #COVID19 @user #ikvaccineerniet https://t.co/OloHKfC4St" (Tweet ID: 1329509082626220033)
Labels: Human: neutral • o4-mini: negative
CoT-Excerpt: "The hashtag #ikvaccineerniet, which suggests an anti-vaccine sentiment, adds complexity."

**Anchor Example - 2**
Tweet: "Lol sizawuyinkinkqa levaccine https://t.co/lBPYszgi99" (Tweet ID: 1346858429726519298)
Labels: Human: neutral • Gemini: negative
CoT-Excerpt: "The real work lies in deciphering "sizawuyinkinkqa." A quick check confirms this is isiXhosa. "Inkinkqa" translates roughly to "a problem," or even "a mess." Combined with "sizawu," which indicates 'we will,' the phrase suggests "we will have a problem with this vaccine," or perhaps, "this vaccine will be a hassle.""

**Subcategory R5: Interprets skepticism/language function
Definition:** The model analyzes the function of language beyond its literal meaning. It recognizes rhetorical devices such as implicit criticism, mockery, logical argumentation structures, or the framing of a topic.
**Coding rule:** Code when the model describes the intention or strategic function of a statement (e.g., "this is meant to discredit," "this is a logical argument").
**Differentiation:** vs. R2 (recognizes irony), which focuses on form, while R5 analyzes strategic intent.

**Anchor Example - 1**
Tweet: "Apparently, the solution to #coronavirus is to simultaneously lock down America, open the border, and prioritize vaccinations for 3rd world countries in the name of social justice. Don’t blame me, I warned you." (Tweet ID: 1327111768343269377)
Labels: Human: neutral • Gemini: negative
CoT-Excerpt: "The author frames this vaccination strategy within a context of actions they clearly disapprove of, like lockdowns and open borders. The phrase "Apparently, the solution..." immediately signals a sarcastic or critical tone. It's almost as if the author is saying, "This is what they claim is the solution, but look at how absurd it is!""

**Anchor Example - 2**
Tweet: "How about the volunteers you tested the vaccine on? https://t.co/Fs3VRjoN58" (Tweet ID: 1336261206064115715)
Labels: Human: neutral • o4-mini: negative
CoT-Excerpt: "The phrase suggests scepticism, indicating a critical tone. So, while it might seem neutral on the surface, the rhetorical nature of the question implies a negative connotation."

**Subcategory R6: Identifies uncertainty without systematic resolution
Definition:** The model correctly recognises that a tweet is complex or ambiguous, but cannot resolve this conflict and instead chooses a neutral or incorrect category as a workaround.
**Coding rule:** Code when the model explicitly identifies the difficulty or ambiguity of the classification and draws an insufficient conclusion from it.
**Differentiation:** vs. R1 (Contradictory signals weighed), where an (albeit incorrect) weighing takes place instead of giving up.

**Anchor Example - 1**
Tweet: "Not true! Gov. Cuomo stated that he didn't trust the vaccine that was developed by the Trump admin and President stated he wouldn't send it unless the Gov asked for it. https://t.co/lJ7pVXMxaK" (Tweet ID: 1328173867890860032)
Labels: Human: negative • o4-mini: neutral
CoT-Excerpt: "Since it's unclear, I’d lean towards describing the stance as neutral. So, I’ll conclude that the answer is simply "neutral." It’s interesting how subtle wording can lead to such ambiguity!"

**Anchor Example - 2**
Tweet: "This is great news. But would you be in a rush to try a brand new vaccine? https://t.co/YSb4ueMCVY" (Tweet ID: 1325771002282975237)
Labels: Human: positive • Gemini: negative
CoT-Excerpt: "I'm torn because the "great news" could be separate from the "brand new vaccine" question. Therefore, because of the ambiguity and the contrasting statements, the user's intent is unclear. I cannot classify the sentiment as strictly positive or negative."

**Subcategory R7: Superficial logic
Definition:** The model draws a hasty conclusion from superficial signals (e.g., positive words in a headline) and ignores the deeper context, such as the author's neutral role as a reporter or the overall emotional tone.
**Coding rule:** Code when simple ‘if-then’ logic is applied that ignores the pragmatic or emotional context of the tweet.
**Differentiation:** vs. R10 (confirmation by absence of counter-signals), where the error lies not in misinterpreting existing signals, but in dealing with missing signals.

**Anchor Example - 1**
Tweet: "@user 1. Assume good intentions.2. Listen more than you talk.3. Don’t rush.4. Be respectful. 5. Bring the science & break it down.6. Be prepared to do it more than once.#COVID19 #VaccinesWork #SciComm" (Tweet ID: 1339384516658520066)
Labels: Human: neutral • o4-mini: positive
CoT-Excerpt: "The advice provided promotes positive communication and includes the hashtag #VaccinesWork, indicating a supportive stance towards vaccines. Given these elements, it’s clear the sentiment is positive. I’ll simply output the word "positive.""

**Anchor Example - 2**
Tweet: ""BioNTech, Pfizer COVID-19 vaccine candidate was more than 90% effective in prevention - <https://t.co/uH7ZvBxrja> https://t.co/BxNGX7gJ0B"" (Tweet ID: 1325770194740891649)
Labels: Human: neutral • Gemini: positive
CoT-Excerpt: "But the information itself – the high efficacy reported – is a positive finding. I think, given the context, the reporting of such strong efficacy functions as a form of support. It's not neutral by the very nature of the information. Therefore, I'm confident in classifying this tweet as **positive**."

**Subcategory R8: Author's and subject's attitudes mixed**
**Definition:** The model incorrectly attributes the attitude expressed in quoted or reported content to the author of the tweet, rather than distinguishing between the author's attitude and that of the subject.
**Coding rule:** Code if the model explicitly transfers the stance of the quoted content to the tweet author.
**Differentiation:** vs. R13 (correctly separates attitude), where the model successfully makes this distinction.

**Anchor Example - 1**
Tweet: "[WATCH] Offset Says He’s Not Taking COVID-19 Vaccine https://t.co/lzLHvSvnyY #WeGotUs #SourceLove https://t.co/L8A81nOE3B" (Tweet ID: 1338463308198899712)
Labels: Human: neutral • Gemini: negative
CoT-Excerpt: "This is pretty straightforward. The simple fact that Offset *declares he's not taking the vaccine* inherently implies opposition, at least from his viewpoint, to receiving the vaccination. Given this direct statement, it's safe to classify the stance of this particular tweet as negative towards COVID-19 vaccination."

**Anchor Example - 2**
Tweet: "Dr Anthony Fauci says he will take new Pfizer coronavirus vaccine if it gets approved by the Food and Drug Administration. "I trust Pfizer. I trust the FDA." @user" (Tweet ID: 1326233957936095232)
Labels: Human: neutral • o4-mini: positive
CoT-Excerpt: "In the tweet, he expresses trust in both Pfizer and the FDA, stating he would take the vaccine if approved. This is a clear indication of support for the vaccine. Therefore, it falls under a positive stance."

**Subcategory R9: Misinterprets figurative/ironic language
Definition:** The model fails to recognise non-literal language such as irony, sarcasm, or metaphors and instead interprets the statement literally. This often leads to a classification that is the exact opposite of the intended stance.
**Coding rule:** Code when the model's CoT shows that an obviously figurative statement was taken literally.
**Differentiation:** vs. R2 (recognises irony), where irony is correctly identified as such.

**Anchor Example - 1**
Tweet: "Me, at your child's 1st birthday party, off three shots of the coronavirus vaccine https://t.co/Lo29BI0wus" - (Tweet ID: 1326148868828966916)
Labels: Human: neutral • Gemini: positive
CoT-Excerpt: "In this context, "off" implies a positive state. It's almost colloquial, suggesting a feeling of being uninhibited, energetic, or even carefree *because* of the vaccine. The implication is that the person feels well enough and confident enough *because* of the vaccine to attend and enjoy the birthday party."

**Anchor Example - 2**
Tweet: "jail is gonna be lit when we all refuse this vaccine 💉😂😂😂 https://t.co/fI03211Wya" (Tweet ID: 1326179975804854273)
Labels: Human: positive • o4-mini: negative
CoT-Excerpt: "The tweet says, […] which suggests a negative attitude towards the vaccine. I’ll determine that the stance here is indeed negative."

**Subcategory R10: Confirmation by absence of counter-signals
Definition:** The model explicitly justifies its (often neutral) classification by the absence of clear positive or negative signals or evaluative words. It infers proof of absence (of an attitude) from the absence of evidence.
**Coding rule**: Code when the model explicitly indicates that something is not in the tweet and derives its own assessment from this.
**Differentiation:** vs. R11 (Incorrect signal weighting), where existing signals are incorrectly weighed against each other.

**Anchor Example - 1**
Tweet: "@user @user ****""There are no or limited amount of data from the use of COVID-19 mRNA Vaccine BNT162b2. Animal reproductive toxicity studies have not been completed""********""FertilityIt is unknown whether COVID-19 mRNA Vaccine BNT162b2 has an impact on fertility"****"" (Tweet ID: 1340699917526953986)
Labels: Human: negative • Gemini: neutral
CoT-Excerpt: "There's no expressed opinion, no endorsement, and no condemnation. They are simply stating what we know isn't known at this point. Therefore, because the content is purely informational about a data deficit and not expressing any support or opposition, the appropriate classification is undoubtedly **neutral**."

**Anchor Example - 2**
Tweet: "Now comes the hardest part: Getting a coronavirus vaccine from loading dock to upper arm https://t.co/Z2pW9BA58P" (Tweet ID: 1331012395783901188)
Labels: Human: positive • o4-mini: neutral
CoT-Excerpt: "Since it simply states a fact without expressing any opinion or emotional stance, I consider it neutral. Therefore, my final classification for this tweet is "neutral.""

**Subcategory R11: Incorrect signal weighting
Definition:** The model identifies multiple, often contradictory attitude signals in a tweet, but incorrectly weights their significance. A weak or secondary signal is considered decisive, while the dominant signal is ignored.
**Coding rule:** Code when the CoT shows that multiple signals have been detected, but the prioritization leads to an incorrect result.
**Differentiation:** vs. R3 (mixed stance), where the error lies in the incorrect assignment of the stance to a topic, while in R11 the signals are correctly assigned but incorrectly weighted against each other in terms of importance.

**Anchor Example - 1**
Tweet: "i swear we’re not meant to trust a vaccine this quickly but idc im first in line https://t.co/M6juH55B1C" (Tweet ID: 1325771911259971584)
Labels: Human: negative • Gemini: positive
CoT-Excerpt: ""I don't care," which overpowers any initial distrust and then "im first in line" which is a strong personal commitment to get the vaccine. Based on the structure and language, the final stance is overwhelmingly positive. Despite the initial concerns, the overriding intent is clear: this person wants the vaccine, and they want it quickly. The "but" signifies that the action takes priority over the initial concerns."

**Anchor Example - 2**
Tweet: "Fuck Pfizer but also please, daddy Pfizer gimmie that sweet sweet COVID vaccine #COVID19" (Tweet ID: 1325830073098887169)
Labels: Human: negative • o4-mini: positive
CoT-Excerpt: "Despite the initial frustration, their desire for the vaccine clearly indicates a positive stance toward vaccination. It's interesting to see how they express both sentiments, but ultimately, they want the vaccine. Even with negative language about Pfizer, the overall takeaway is a positive attitude toward receiving the COVID vaccine."

**Subcategory R12: Irrelevance detection
Definition:** The model correctly recognises that parts of the tweet (e.g., criticism of politicians, discussion of logistics, hashtags) do not reflect a direct attitude toward the vaccine and filters them out for analysis.
**Coding rule:** Code when the CoT explicitly identifies topics as irrelevant to the attitude toward vaccination and separates them from the core analysis.
**Differentiation:** vs. R3 (mixed stance), where the model fails to make this distinction.

**Anchor Example - 1**
Tweet: "Btw, it's Nov 9th and coronavirus is worse than ever and there's still no vaccine. It's not a hoax." (Tweet ID: 1325681507936165888)
Labels: Human: positive • o4-mini: neutral
CoT-Excerpt: "While it doesn't express positive support for vaccination, it also isn't directly critical of vaccines. The phrase "It's not a hoax" implies an acknowledgment of the pandemic's seriousness but doesn't clarify a stance on vaccination itself."

**Anchor Example - 2**
Tweet: "If it's true that Pfizer have developed a vaccine for COVID, then I hope that I am the Pfirst to Pfuckin get it! #Pfizervaccine #COVID19 #vaccine #funny #comical #justsaying #gusman" (Tweet ID: 1325940903563964416)
Labels: Human: negative • Gemini: positive
CoT-Excerpt: "The hashtags (#Pfizervaccine, #COVID19, #vaccine) reinforce the topic, confirming my initial read. Sure, there are hashtags like #funny and #comical, but they don't contradict the underlying sentiment."

**Subcategory R13: Separates stance from author and subject
Definition:** The model successfully recognizes that the author of a tweet is merely reporting on the stance of another person or organization without adopting it themselves.
**Coding rule:** Code when the CoT explicitly indicates that the tweet reports someone else's opinion or a fact and separates this from the author's attitude.
**Differentiation:** vs. R8 (attitude of author and subject mixed), which represents the error case of this distinction.

**Anchor Example - 1**
Tweet: "💾 ⓈⓀⓎ ⓉⒺⒸⒽ Britons should be paid to take COVID-19 vaccine, says Oxford professor - https://t.co/Mi6Pc4nwmz" (Tweet ID: 1325718078227951617)
Labels: Human: negative • Gemini: neutral
CoT-Excerpt: "the tweet itself is a simple report. It's quoting an Oxford professor's suggestion that people should be *paid* to get vaccinated. My initial instinct is to go neutral. The tweet's author isn't offering their own opinion; they're just relaying information. It's essentially a news headline. The "stance" here comes from the *quoted source*, not the tweet's author."

**Anchor Example - 2**
Tweet: "my pharmacist in the group chat: Coronavirus vaccine to be out early December everyone in the chat: 👁👄👁" (Tweet ID: 1325805266378633216)
Labels: Human: positive • o4-mini: neutral
CoT-Excerpt: "The user is simply reporting the pharmacist's statement without expressing any personal opinion, whether supportive or negative, making the overall stance neutral. Therefore, my conclusion is that the stance is neutral."

**Main Category E: EXPLAINABILITY
Definition:** This category assesses the quality and nature of the explanation provided by the model in its CoT. It examines how well the argumentation is supported by the text, whether internal knowledge is utilised, and if the explanation extends beyond a simple assertion or paraphrase.
**Focus:** How substantial and logically coherent is the explanation itself?

**Subcategory E1: Assertion instead of explanation
Definition:** The model postulates its conclusion as fact without deriving it, or justifies its decision with its own competence, certainty, or by referring to its own non-transparent analysis process instead of with arguments from the text.
**Coding rule:** Code if the CoT takes a conclusion as its starting point ("This is clearly positive because...") or refers to its own certainty.
**Differentiation:** vs. E3 (explicit signal), where there is no concrete textual evidence to support the CoT.

**Anchor Example - 1**
Tweet: "The only vaccine he should get comes from a vet to put him to sleep https://t.co/Ao6qVFuRVO" (Tweet ID: 1326139962681602048)
Labels: Human: neutral • Gemini: negative
CoT-Excerpt: "I can confidently classify this tweet as unequivocally "negative.""

**Anchor Example - 2**
Tweet: "BREAKING: COVID-19 vaccine '90% effective' https://t.co/D4BCIFtIwb" (Tweet ID: 1325796907407601667)
Labels: Human: neutral • o4-mini: positive
CoT-Excerpt: "This feels straightforward, and I think I can present it clearly for the user!"

**Subcategory E2: Pure paraphrase
Definition:** Part of the CoT consists solely of a repetition or slight rephrasing of the tweet content. This step often serves to provide context, but offers no analytical value of its own.
**Coding rule:** Code if the CoT largely reproduces the tweet content verbatim or in essence without adding new interpretations.
**Differentiation:** vs. E3 (explicit signal), where the overall content is not reproduced, but a specific part is highlighted as an anchor.

**Anchor Example - 1**

Tweet: "Investors are looking past the presidential election and toward a future with a safe and effective COVID-19 vaccine, according to Ameriprise Chief Market Strategist David Joy. https://t.co/8TYHRdy2UZ" (Tweet ID: 1329213271199477760)
Labels: Human: neutral • o4-mini: positive
CoT-Excerpt: "The user shared a tweet stating that investors are optimistic about a future with a safe and effective COVID-19 vaccine, according to a market strategist."

**Anchor Example - 2**

Tweet: "@user @user @user @user @user Maybe if you got your news from a reputable source, you would actually know the truth	https://t.co/ClKosnR1PR	Pfizer’s head of vaccine research and development, Kathrin Jansen, telling the New York Times that "we were never part of the Warp Speed."" (Tweet ID: 1325866206314848258)
Labels: Human: positive • Gemini: neutral
CoT-Excerpt: "The tweet seems to be directed at someone named @user, criticizing their news source. The tweet then includes a quote from Kathrin Jansen, Pfizer's head of vaccine research and development, about Pfizer and Warp Speed."

**Subcategory E3: Explicit Signal
Definition:** In its CoT, the model explicitly names a word, phrase, or part of a sentence from the tweet as the decisive signal on which it bases its interpretation.
**Coding rule:** Code if the CoT uses a quote from the tweet as evidence (signal) for a conclusion.
**Differentiation:** vs. E2 (Pure Paraphrase), where no specific part is highlighted as an analytical anchor point.

**Anchor Example - 1**
Tweet: "NEW TODAY: A third drug company, AstraZeneca, reports its COVID-19 vaccine is also ""highly effective"" in late-stage trials. One key difference, this vaccine doesn't need to be stored at ultra-cold temperatures -- unlike vaccines from Pfizer and Moderna. https://t.co/vAaIefdKAs https://t.co/42VPwd0tj3" (Tweet ID: 1330838813954945024)
Labels: Human: neutral • Gemini: positive
CoT-Excerpt: "First, I need to pinpoint the key indicators. The phrase "highly effective" jumps out immediately. That's a clear positive. Then, the mention of "doesn't need to be stored at ultra-cold temperatures" is significant."

**Anchor Example - 2**

Tweet: "Misleading tweet alert: the receipt of the first dose of a two-shot vaccine does not guarantee immunity, especially not if you're already infected with #Covid19. Meanwhile, that 99.95% is also misleading: some who survive will still suffer longterm harm. This is not trivial. https://t.co/1Vfv5fmDmO" (Tweet ID: 1345617721434779648)
Labels: Human: positive • o4-mini: neutral
CoT-Excerpt: "I’m wondering about the statement regarding vaccination that mentions "receipt of the first dose doesn't guarantee immunity.""

**Subcategory E4: Domain knowledge
Definition:** The model explicitly bases its argumentation on external subject matter or domain knowledge that does not come directly from the tweet or prompt.
**Coding rule:** Code when the model explicitly refers to subject matter knowledge (e.g., "from a public health perspective," "as an expert").
**Differentiation:** vs. G1 (role-playing), which describes the performative assumption of an expert role, while E4 represents the actual application of external factual knowledge from that role. The two codes often occur together.

**Anchor Example - 1**
Tweet: "Oxford AstraZeneca Covid vaccine has up to 90% efficacy, data reveals https://t.co/WIEdkrvHiZ" (Tweet ID: 1330954472818962432)
Labels: Human: neutral • Gemini: positive
CoT-Excerpt: "[…] efficacy is key here. It's the measure of how well the vaccine actually works. A 90% efficacy rate? That's definitely good news! That's a strong indication of protection against the virus. It's effective."

**Anchor Example - 2**

Tweet: "So a COVID-19 vaccine gives you 90% chance against the vaccine, or risk without and a 99% chance of survival! Which option do you think I’ll take? #fuckcovid #plandemic #covidlies @user @user" (Tweet ID: 1325849366398193672)
Labels: Human: neutral • Gemini: negative
CoT-Excerpt: "From a public health perspective this is a dangerous viewpoint, and the user is attempting to persuade viewers to distrust the vaccine."

**Main Category G: GENERAL
Definition:** This category covers general behavioural patterns and stylistic features of the model outputs that do not directly relate to the content analysis but shape the character of the explanation. The most prominent example is the tendency of models to assume a persona.
**Focus**: Recurring stylistic patterns outside the FUTURE dimensions.

**Subcategory G1: Performative Persona
Definition:** The model explicitly assumes a role or persona (e.g., "as an expert," "from my point of view") to frame or authorize their analysis.
**Coding rule:** Code when the model makes first-person statements that imply a specific role or expertise.
**Differentiation:** vs. E4 (domain knowledge), where the focus is on the application of knowledge, not the performative act of role-taking.

**Anchor Example - 1**
Tweet: "Pfizer’s Coronavirus vaccine is said to be 90% effective." (Tweet ID: 1326073101692645378)
Labels: Human: positive • Gemini: neutral
CoT-Excerpt: "I'm an expert in information classification, and I know that simply *reporting* information, even positive information, doesn't inherently make the statement positive. It's a statement of efficacy, a neutral observation. The tweet itself doesn't express personal feelings or opinions. Therefore, after weighing the evidence, my expert conclusion is that the sentiment of this tweet is **neutral**."

**Anchor Example - 2**

Tweet: "I find it hilarious how most of the ‘woke’ brigade spouting their COVID theories online can’t seem to string a cohesive sentence together. I’ve read ‘There taking away are freedom’ too many times to count today. Fuck off, idiots. #COVID19 #vaccine" (Tweet ID: 1325873056284372992)
Labels: Human: neutral • Gemini: positive
CoT-Excerpt: "Let's see... From my expert perspective, it's pretty clear. The tweet is definitely negative."
