# Supplementary material for: Beyond Accuracy: A Mixed-Methods Audit of Chain-of-Thought Failures in LLM-Based COVID-19 Vaccine Stance Detection
Source: J Med Syst. 2026 Aug 8;50(1):123. doi: 10.1007/s10916-026-02449-3 (PMC13452790; doi:10.1007/s10916-026-02449-3)
Supplement: Supplementary file 5 [file 10916_2026_2449_MOESM5_ESM.docx]

**Supplementary material for:**
Beyond accuracy: a mixed-methods audit of chain-of-thought failures in LLM-based COVID-19 vaccine stance detection.

**Journal:** Journal of Medical Systems

Andreas Praschk, BSc; Valentin Fischill-Neudeck, PhD, MScN; Thomas Caspari, Dr rer nat; Hans-Peter Wiesinger, Dr rer nat, LLB oec

Paracelsus Medical Private University, Center for Public Health and Healthcare Research, Strubergasse 21, 5020 Salzburg, Austria

Corresponding author: Hans-Peter Wiesinger, E-mail: hans-peter.wiesinger@pmu.ac.at

**Table 6** Coverage and dual-error breakdown for tweets with CoT in both models

| **Metric** | **n (%)** |
| --- | --- |
| Tweets with CoTs in **both** models | 1981 |
| **Dual-errors**: both models wrong (vs. human) | 295 (14.89% of 1981) |
| └─ of dual-errors: same wrong label (model-model agreement on wrong class) | 262 (88.81% of 295) |
| ⤷ neutral → positive (both models) | 102 (38.9% of 262) |
| ⤷ neutral → negative | 57 (21.8% of 262) |
| ⤷ positive → neutral | 40 (15.3% of 262) |
| ⤷ positive → negative | 31 (11.8% of 262) |
| ⤷ negative → neutral | 17 (6.5% of 262) |
| ⤷ negative → positive | 15 (5.7% of 262) |
| └─ of dual-errors: different wrong labels | 33 (11.19% of 295) |

*Notes: The CoT subset includes tweets for which both models produced a non-missing, non-"<no summary>" reasoning_summary (N = 1,981). "Dual-errors" are items misclassified by both models relative to the human-annotated reference label. Percentages are with respect to the parent row; the "same wrong label" and "different wrong labels" rows are computed within the dual-error subset; the direction rows (reference label → shared wrong label) are computed within the 262 same-wrong-label cases. These 295 tweets formed the sampling frame for the qualitative audit of dual-error cases (see Methods). The proportion of dual-error cases with the same wrong label was 88.8% (262/295); a percentile bootstrap (B = 10,000; seed = 42) yielded a 95% confidence interval of 85.1–92.2%.*
