# Supplementary material for: Beyond Accuracy: A Mixed-Methods Audit of Chain-of-Thought Failures in LLM-Based COVID-19 Vaccine Stance Detection
Source: J Med Syst. 2026 Aug 8;50(1):123. doi: 10.1007/s10916-026-02449-3 (PMC13452790; doi:10.1007/s10916-026-02449-3)
Supplement: Supplementary file 6 [file 10916_2026_2449_MOESM6_ESM.docx]

**Supplementary material for:**
Beyond accuracy: a mixed-methods audit of chain-of-thought failures in LLM-based COVID-19 vaccine stance detection.

**Journal:** Journal of Medical Systems

Andreas Praschk, BSc; Valentin Fischill-Neudeck, PhD, MScN; Thomas Caspari, Dr rer nat; Hans-Peter Wiesinger, Dr rer nat, LLB oec

Paracelsus Medical Private University, Center for Public Health and Healthcare Research, Strubergasse 21, 5020 Salzburg, Austria

Corresponding author: Hans-Peter Wiesinger, E-mail: hans-peter.wiesinger@pmu.ac.at

**Per-tweet predictions and qualitative codes**

The accompanying data file (ESM7_PredictionsAndCodes.csv, semicolon-separated) provides one row per analysed tweet (N = 3,060), keyed by tweet ID. In line with X’s Developer Policy, tweet text is not redistributed. Columns:

– tweet_id: Tweet identifier (rehydratable via the X API)

– gold_label: Human-annotated reference stance label (positive / negative / neutral) from Cotfas et al.

– o4mini_low_pred: o4-mini prediction at low reasoning effort (primary performance configuration)

– o4mini_high_pred: o4-mini prediction at high reasoning effort (audit configuration)

– gemini250_pred: Gemini 2.5 Flash prediction at thinking budget 250 (primary and audit configuration)

– o4mini_high_has_cot: 1 if o4-mini (high) returned a reasoning summary, else 0

– gemini250_has_cot: 1 if Gemini (250) returned a reasoning summary, else 0

– is_dual_error: 1 if the tweet belongs to the 295 dual-error cases (both audit-configuration predictions wrong among the 1,981 tweets with a reasoning summary from both models), else 0

– n_T1_coder1 to n_G1_coder1: Number of coded segments per failure-mode subcode assigned by the first coder (23 columns; dual-error cases only)

– n_T1_coder2 to n_G1_coder2: Number of coded segments per subcode assigned by the second coder, who independently re-coded all 295 dual-error cases (23 columns)

Note on code counts: The count columns record segment-level frequencies, so a subcode may be counted several times within one case; the subcode frequencies in Figure 1 of the main text equal the column sums of the first coder. A subcode was assigned at case level if its count is greater than zero; the inter-coder agreement statistics reported in Online Resource 8 were computed on these case-level values. Count cells are filled for the 295 dual-error cases only and left empty for all other tweets.
