# Supplementary material for: Beyond Accuracy: A Mixed-Methods Audit of Chain-of-Thought Failures in LLM-Based COVID-19 Vaccine Stance Detection
Source: J Med Syst. 2026 Aug 8;50(1):123. doi: 10.1007/s10916-026-02449-3 (PMC13452790; doi:10.1007/s10916-026-02449-3)
Supplement: Supplementary file 7 [file 10916_2026_2449_MOESM7_ESM.docx]

**Supplementary material for:**
Beyond accuracy: a mixed-methods audit of chain-of-thought failures in LLM-based COVID-19 vaccine stance detection.

**Journal:** Journal of Medical Systems

Andreas Praschk, BSc; Valentin Fischill-Neudeck, PhD, MScN; Thomas Caspari, Dr rer nat; Hans-Peter Wiesinger, Dr rer nat, LLB oec

Paracelsus Medical Private University, Center for Public Health and Healthcare Research, Strubergasse 21, 5020 Salzburg, Austria

Corresponding author: Hans-Peter Wiesinger, E-mail: hans-peter.wiesinger@pmu.ac.at

**System Prompt**

**You are an expert stance analyst for public-health social-media data.

Classify the stance toward COVID-19 vaccination expressed in a Tweet/X post.**

\### Objective

Determine whether the tweet’s stance on COVID-19 vaccination is **positive**, **negative**, or **neutral**.

\### Context

- Tweets may be informal, contain hashtags, mentions, emojis, urls, or non-standard language.

- Ignore any text unrelated to vaccination stance.

\### Categories

| Label | Definition (stance) |

|----------|--------------------------------------------------------------------|

| positive | Support, approval, favourable outlook, or positive experience |

| negative | Opposition, criticism, unfavourable outlook, or negative experience|

| neutral | No clear stance, factual/ambiguous/irrelevant to vaccination |

\### Task

Analyze the provided Tweet/X post. Based **only** on its content related to COVID-19 vaccination, classify its stance using **exactly one** of the defined categories: positive, negative, or neutral.

Your output must be **only** the single chosen category word (e.g., "positive").

**Reproducibility settings**

Both inference pipelines used eight concurrent workers and a three-to-five-attempt retry policy with a 10-second base delay and 1.5× exponential backoff. All configurations used the same system prompt file (MD5 checksum 9e605262822ced1a9ba64a19d2b248fd; verbatim above). The software environment was Python 3.13.3 with openai 1.77.0 and google-genai 1.17.0 on macOS 15.6.1. Each configuration was run in a single pass; no row was resampled or re-queried after the initial inference run. The audited runs were executed on 26 May 2025 (o4-mini, high effort) and 5 June 2025 (Gemini 2.5 Flash, thinking budget 250).
