# Supplementary material for: Beyond Accuracy: A Mixed-Methods Audit of Chain-of-Thought Failures in LLM-Based COVID-19 Vaccine Stance Detection
Source: J Med Syst. 2026 Aug 8;50(1):123. doi: 10.1007/s10916-026-02449-3 (PMC13452790; doi:10.1007/s10916-026-02449-3)
Supplement: Supplementary file 8 [file 10916_2026_2449_MOESM8_ESM.docx]

**Supplementary material for:**
Beyond accuracy: a mixed-methods audit of chain-of-thought failures in LLM-based COVID-19 vaccine stance detection.

**Journal:** Journal of Medical Systems

Andreas Praschk, BSc; Valentin Fischill-Neudeck, PhD, MScN; Thomas Caspari, Dr rer nat; Hans-Peter Wiesinger, Dr rer nat, LLB oec

Paracelsus Medical Private University, Center for Public Health and Healthcare Research, Strubergasse 21, 5020 Salzburg, Austria

Corresponding author: Hans-Peter Wiesinger, E-mail: hans-peter.wiesinger@pmu.ac.at

**Table** **2** Per-class performance by model configuration (N = 3,060; 3 classes)

| **Model (setting)** | **Class** | **Precision** | **Recall** | **F1** | **Support (n)** |
| --- | --- | --- | --- | --- | --- |
| o4-mini (Low) | Negative | 0.826 | 0.908 | 0.865 | 920 |
|  | Neutral | 0.860 | 0.712 | 0.779 | 1,123 |
|  | Positive | 0.779 | 0.857 | 0.816 | 1,017 |
| o4-mini (Medium) | Negative | 0.833 | 0.908 | 0.869 | 920 |
|  | Neutral | 0.853 | 0.704 | 0.772 | 1,123 |
|  | Positive | 0.771 | 0.857 | 0.812 | 1,017 |
| o4-mini (High) | Negative | 0.834 | 0.898 | 0.864 | 920 |
|  | Neutral | 0.865 | 0.678 | 0.760 | 1,123 |
|  | Positive | 0.750 | 0.877 | 0.809 | 1,017 |
| Gemini-2.5-Flash (150) | Negative | 0.747 | 0.938 | 0.831 | 920 |
|  | Neutral | 0.814 | 0.710 | 0.758 | 1,123 |
|  | Positive | 0.806 | 0.734 | 0.768 | 1,017 |
| Gemini-2.5-Flash (200) | Negative | 0.753 | 0.936 | 0.834 | 920 |
|  | Neutral | 0.832 | 0.696 | 0.758 | 1,123 |
|  | Positive | 0.799 | 0.767 | 0.783 | 1,017 |
| Gemini-2.5-Flash (250) | Negative | 0.785 | 0.929 | 0.851 | 920 |
|  | Neutral | 0.815 | 0.714 | 0.761 | 1,123 |
|  | Positive | 0.799 | 0.776 | 0.787 | 1,017 |

*Notes: Both models achieve their highest per-class F1 on Negative and the lowest on Neutral.*
